# Finding neural assemblies with frequent item set mining

**DOI:** 10.3389/fninf.2013.00009

**Published:** 2013-05-31

**Authors:** David Picado-Muiño, Christian Borgelt, Denise Berger, George Gerstein, Sonja Grün

**Affiliations:** ^1^European Centre for Soft Computing, Calle Gonzalo Gutiérrez Quirós s/nMieres, Asturias, Spain; ^2^Laboratory of Neuromotor Physiology, IRCCS Fondazione Santa LuciaRoma, Italy; ^3^Department of Neuroscience, University of PennsylvaniaPhiladelphia, PA, USA; ^4^Institute of Neuroscience and Medicine (INM-6) and Institute for Advanced Simulation (IAS-6), Jülich Research Centre and JARAJülich, Germany; ^5^Theoretical Systems Neurobiology, RWTH Aachen UniversityAachen, Germany; ^6^Cognitive Brain Mapping, RIKEN Brain Science InstituteWako-Shi, Japan

**Keywords:** massively parallel spike trains, cell assembly, synchronous spike patterns, higher-order correlation, frequent item set mining, surrogate data, multi-variate significance testing

## Abstract

Cell assemblies, defined as groups of neurons exhibiting precise spike coordination, were proposed as a model of network processing in the cortex. Fortunately, in recent years considerable progress has been made in multi-electrode recordings, which enable recording massively parallel spike trains of hundred(s) of neurons simultaneously. However, due to the challenges inherent in multivariate approaches, most studies in favor of cortical cell assemblies still resorted to analyzing pairwise interactions. However, to recover the underlying correlation structures, higher-order correlations need to be identified directly. Inspired by the Accretion method proposed by Gerstein et al. (1978) we propose a new assembly detection method based on frequent item set mining (FIM). In contrast to Accretion, FIM searches effectively and without redundancy for individual spike patterns that exceed a given support threshold. We study different search methods, with which the space of potential cell assemblies may be explored, as well as different test statistics and subset conditions with which candidate assemblies may be assessed and filtered. It turns out that a core challenge of cell assembly detection is the problem of *multiple testing*, which causes a large number of *false discoveries*. Unfortunately, criteria that address individual candidate assemblies and try to assess them with statistical tests and/or subset conditions do not help much to tackle this problem. The core idea of our new method is that in order to cope with the multiple testing problem one has to shift the focus of statistical testing from specific assemblies (consisting of a specific *set* of neurons) to spike patterns of a certain size (i.e., with a certain *number* of neurons). This significantly reduces the number of necessary tests, thus alleviating the multiple testing problem. We demonstrate that our method is able to reliably suppress false discoveries, while it is still very sensitive in discovering synchronous activity. Since we exploit high-speed computational techniques from FIM for the tests, our method is also computationally efficient.

## 1. Introduction

The principles of neural information processing are still under intense debate. Although changes in the firing rates of individual neurons are observed in relation to stimuli and behavior, the role of these changes in the joint information processing executed by *networks* of neurons is not yet clear.

As a model of network processing, cell assemblies were proposed (Hebb, 1949), which are characterized as groups of neurons exhibiting precise spike coordination. Since it can be shown theoretically that synchronous firing is most effective in generating output spikes of downstream neurons (Abeles, 1982; König et al., 1996; Schultze-Kraft et al., 2013), the *synfire chain* was proposed as a more specific model of cortical activity (Abeles, 1991). Experimental evidence from correlation analyses showed that spike synchrony indeed occurs and in particular in relation to behavior and learning (e.g., Freiwald et al., 1995; Vaadia et al., 1995; Riehle et al., 1997; Kilavik et al., 2009). However, these studies were limited to fairly small numbers of neurons recorded simultaneously, and thus it was not possible to uncover the (full) underlying correlation structure.

Fortunately, in recent years considerable progress has been made in multi-electrode recordings (e.g., Nicolelis et al., 1997; Buzsaki, 2004), which enable to record the activity of hundred(s) of neurons simultaneously. However, due to the challenges inherent in multivariate approaches (especially the combinatorial explosion of the spike patterns that need to be checked), most studies in favor of cortical cell assemblies still resorted to analyzing pairwise interactions. Although in this way the existence and functional relevance of pairwise interactions could be demonstrated in various cortical systems and behavioral paradigms (e.g., Gerstein and Aertsen, 1985; Kohn and Smith, 2005; Fujisawa et al., 2008; Feldt et al., 2009; Masud and Borisyuk, 2011), which can also be used to discover correlated groups of neurons by subsequent clustering (e.g., Berger et al., 2007; Fujisawa et al., 2008), higher-order correlations need to be identified directly in order to recover the (full) correlation structures.

Higher-order correlations can be addressed on different levels, based on (correlation) statistics of recorded parallel spike trains and corresponding statistical tests, which focus on the following aspects: (1) test whether higher-order spike correlation is present, possibly with a lower bound on the order, but without identifying the participating neurons (e.g., Louis et al., 2010a; Staude et al., 2010a,b); (2) test for individual neurons whether they participate in synchronous spiking activity, but without identifying the groups of correlated neurons (Berger et al., 2010); (3) test for the presence of correlation as predicted by a specific correlation model (synfire chain, Abeles, 1991), that is, spatio-temporal spike patterns or propagation of synchronous spiking activity (e.g., Abeles and Gerstein, 1988; Schrader et al., 2008; Gansel and Singer, 2012; Gerstein et al., 2012); (4) actually identify the members of cell assemblies that exhibit synchronous spiking activity (e.g., Gerstein et al., 1978; Pipa et al., 2008; Feldt et al., 2009; Shimazaki et al., 2012).

In this paper we focus on the last category and solve three major challenges simultaneously: (1) detect and identify members of an active cell assembly directly as significant spike synchrony patterns (2) with an efficient and reliable statistical method that (3) is applicable to massively parallel spike trains, i.e., in the order of hundred(s) of spike trains or more.

In order to motivate and justify our new method, we study different search methods, with which the space of potential cell assemblies may be explored, and different test statistics and subset conditions with which candidate assemblies may be assessed and filtered. It turns out that a core challenge of cell assembly detection is the problem of *multiple testing*, which causes a large number of *false discoveries*. That is, many neuron groups are reported as cell assemblies that actually are not. Unfortunately, criteria that address individual candidate cell assemblies and try to assess them with statistical tests and/or subset conditions do not help much to tackle this problem.

Based on these results, we propose a new assembly detection method. The core idea of this method is that in order to cope with the multiple testing problem we shift the focus of statistical testing from specific assemblies (consisting of a specific *set* of neurons) to assemblies of a certain size (that is, with a certain *number* of neurons, thus pooling different sets of neurons). This significantly reduces the number of necessary tests, thus alleviating the multiple testing problem. Note, however, that only the focus of the statistical testing is shifted—the assemblies reported by our method are still specific sets. We demonstrate that our method is able to reliably suppress most false discoveries, while still being very sensitive in discovering synchronous activity. Since we exploit high-speed computational techniques from frequent item set mining (FIM) for the tests, our method is also computationally efficient, even if we are faced with hundred(s) of parallel spike trains.

The remainder of this paper is structured as follows: in Section 2 we introduce some notation and preliminaries on time-bin discretization and spike synchrony. In section 3 we briefly review the Accretion methodology (Gerstein et al., 1978), which forms the starting point of our investigation. In section 4 we briefly introduce FIM (Goethals, 2010; Borgelt, 2012), a data mining technique closely related to Accretion, which has a conceptually similar objective. In section 5 we compare Accretion and FIM, reveal the redundancy in Accretion's search scheme, and show how ideas from FIM can be used to eliminate it. Section 6 introduces several assembly detection criteria and how they can be incorporated into FIM approaches. In section 7 these criteria are evaluated on artificially generated data, comparing FIM and Accretion and demonstrating that the detection quality suffers severely from *false discoveries* brought about by *multiple testing*. Building on the insights gained we then introduce our novel methodology in section 8. Finally, we conclude the paper with a discussion of the merits of our method over the previously investigated approaches.

## 2. Notions and notation

Throughout this paper we work with a finite set *N* of neurons. Our raw data is a collection of *N* (simulated) spike trains of total duration *T*, each consisting of a list of spike times in (0, *T*].

In order to characterize and quantify synchrony among simultaneous spike trains even in the presence of temporal imprecision (regardless of whether it stems from the recording equipment or is a feature of the recorded process) we work on discretized spike data (e.g., Grün et al., 2002, “exclusive binning”). That is, we partition the time interval (0, *T*] under consideration into time bins of equal length. Spikes (or, more precisely, spike times) corresponding to distinct neurons in *N*—say, to a subset *A* ⊆ *N*—are considered synchronous (i.e., a *synchronous event for A*, or simply *A-event*) if they lie in the same time bin. The amount of synchrony of a group of neurons *A* ⊆ *N* (i.e., the amount of synchrony observed in the corresponding collection of spike trains) is simply the number of time bins that contain at least one *A*-event (that is, we “clip” bin entries to 1 if more than one spike of one neuron falls into a time bin).

## 3. Accretion

The starting point of our investigation is a statistical technique proposed in Gerstein et al. (1978), which aims at identifying *neural assemblies* (called *functional groups* in Gerstein et al. (1978) and intuitively understood as groups of neurons that tend to show *significant* synchronous spiking) in parallel spike trains. This method is *accretional* in nature, and therefore generally referred to as *Accretion* in the following: sequences of neurons are formed and extended by an iterative application of a statistical independence test between a new neuron and an already accreted sequence of neurons, based on the number of joint spiking events.

Accretion relies on Pearson's χ^2^ *independence test* to assess whether paired observations of two sets *A* and *B* of neurons, as expressed in a contingency table (see Table 1), are independent of each other. The counts in Table 1 range over the number of time bins: for example, *n*_11_ is the number of time bins that contain both an *A*-event and a *B*-event^1^. Formally, Pearson's χ^2^ statistic is defined as follows, with the counts from Table 1, for disjoint sets of neurons *A, B* ⊆ *N*:

$${\text{χ}}^{\text{2}}(A,B)=n_{**}\frac{{\left(n_{1*}n_{*1}-n_{**}n_{11}\right)}^{2}}{n_{1*}n_{0*}n_{*1}n_{*0}}.$$

Accretion considers two sets *A* and *B* as showing significant synchronous spiking activity according to Pearson's χ^2^ test if the null hypothesis of independence can be rejected with a significance level of α = 0.01 = 1%, that is, if χ^2^(*A, B*) ≥ χ^2^_1, 1−α_ ≈ 6.635, where χ^2^_1, 1−α_ is the 1 − α = 99% quantile of the χ^2^ distribution with one degree of freedom. Equivalently, but often more conveniently, we may compute the *p*-value of the χ^2^ test, which is defined by the relation χ^2^_1, 1−*p*_ = χ^2^(*A, B*). In this case the test result is significant if *p* ≤ α.

**Table 1:** **A 2 × 2 contingency table for binary events *A* and *B***.

	**0 (not B)**	**1 (B)**	**Sum**
**0 (not A)**	*n*_00_	*n*_01_	*n*_0*_
**1 (A)**	*n*_10_	*n*_11_	*n*_1*_
**sum**	n_*0_	n_*1_	n_**_

Note, however, that Accretion considers this test result only if *n*_11_n_**_ > *n*_1*_n_*1_, that is, if there are more synchronous spiking events than are to be expected under independence. Otherwise the two (sets of) neurons are regarded as not correlated. The difference to relying entirely on the test result is marginal, though, because of the asymmetry of the distribution.

In its first step, Accretion tests all pairs formed by singletons *A, B* ⊂ *N* (that is, |*A*| = |*B*| = 1) and selects all two-element sequences that show significant synchronous spiking (according to the test criterion described above). Subsequent steps (try to) expand accreted sequences in all possible ways by adding another neuron. That is, in the *n*th step, *n* < |*N*|, sequences formed in the (*n* − 1)th step (that is, sequences of neurons in sets *A* ⊂ *N* with |*A*| = *n*) are expanded by all singletons *B* ⊂ *N* \ *A* (that is, |*B*| = 1) and tested for independence. Those that show significant synchrony (w.r.t. Pearson's χ^2^ test and a chosen significance level α) are selected and possibly expanded further in later steps. Significant sequences that cannot be expanded anymore (because no additional neuron passes a test for significant synchrony with them) are finally reported as (candidates for) neural assemblies.

Note that the original Accretion limits significant expansions to two. That is, the branching factor of the search is two: any significant sequence gives rise to at most two expanded sequences. Although it is tempting to ascribe this constraint to limitations of the computer hardware at the time (1978) and thus to omit it, it is actually still needed today due to the redundant search scheme of Accretion (see below for details). Nevertheless we ignore this branching restriction for the following considerations in order to avoid some technical, but largely irrelevant complications. That is, we assume for now that any additional neuron that passes a synchrony test with an accreted sequence gives rise to an expanded sequence.

Without a branching restriction, we can formalize the implicit characterization of a neural assembly underlying Accretion in terms of *subset conditions*. We say that *A* ⊆ *N* constitutes a *synchronous group* if it satisfies the following:

- If |*A*| = 2 then the two singleton subsets of *A* must show significant synchrony (as evaluated by Pearson's χ^2^ independence test w.r.t. a significance level α, see above).
- If |*A*| > 2 then there has to exist a subset *B* ⊂ *A* with |*B*| = |*A*| − 1 that is a *synchronous group* and that shows significant synchrony with the remaining neuron in *A*, that is, with *A* \ *B* (again as evaluated by Pearson's χ^2^ test). (Note the recursive structure of this definition.)

Accretion reports as neural assemblies only significant sequences that cannot be expanded anymore. That is, it never reports proper prefixes (and thus subsets) of a detected assembly. Hence we may say that Accretion regards as neural assemblies *maximal synchronous groups*. Here “maximal” expresses that there does not exist a superset that is also a synchronous group.

**Figure 1:**
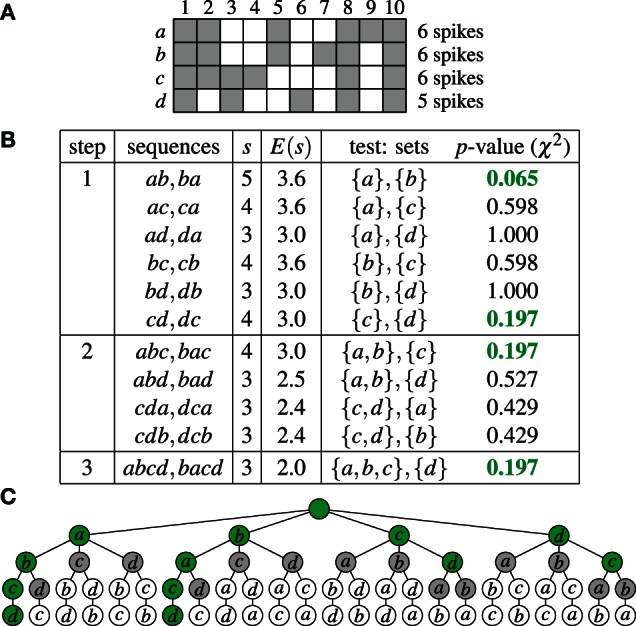
**Accretion example: (A)** Spikes of four neurons *N* = {*a, b, c, d*} in 10 time bins. A gray square indicates the presence of a spike in an time bin. **(B)** Accretion for the data shown in **(A)** for a significance level α = 0.2. Column *s* states the actual, column *E*(*s*) the expected number of joint spiking events under independence. The *p*-values shown in green are significant. **(C)** Graphical representation of the process shown in **(B)** in the search space. Selected paths are marked in green.

As an example, Figure 1A shows an extremely simple binned parallel spike train for four neurons *a, b, c*, and *d* (that is, *N* = {*a, b, c, d*}) and 10 time bins. For each time bin the neurons having a spike in it are marked. Figure 1B shows how this data is processed by Accretion in three steps, using a significance level α = 0.2. (Clearly, due to the very low number of time bins, no significant results could be obtained for α = 0.01.) As a result, Accretion reports the four sequences *abcd, bacd, cd* and *dc*, which are the maximal synchronous groups.

As a further illustration Figure 1C shows the complete search space of the Accretion method for this example, that is, all sequences that are potentially explored. The green nodes correspond to significant test results (with the empty sequence—root node—and the singletons being considered significant by default). Gray nodes are visited, but do not yield significant test results. White nodes are not visited. Accretion reports the sequences corresponding to the deepest green nodes, listing all neurons on the path from the root to these nodes.

## 4. Frequent item set mining

FIM was originally motivated by the desire to find regularities in the shopping behavior of customers (of supermarkets, mail-order companies, on-line shops etc.) by identifying sets of products that are frequently bought together [*market basket analysis*, see the seminal paper (Agrawal et al., 1993) and the surveys (Goethals, 2010; Borgelt, 2012)]. Conceptually, this task is obviously the same as finding sets of neurons that (frequently) fire together in parallel spike trains, which establishes the relevance of FIM for detecting neural assemblies in parallel spike trains.

Formally, we consider an item base *I* = {*i*_1_,…, *i*_*n*_}, *n* ∈ ℕ, and a database 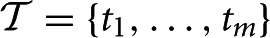 of transactions, *m* ∈ ℕ, where *t*_*k*_ (the *k* th transaction) is a pair 〈*k, J*_*k*_〉 consisting of a unique identifier *k* and a subset of items *J*_*k*_ ⊆ *I*. In our context the item base *I* is the set *N* of neurons and the transactions are determined by the time bins into which we partition the recording time interval (0, *T*]: the set of neurons with spike times lying in the *k* th time bin constitutes *J*_*k*_ in the *k* th transaction 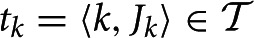. Table 2 summarizes the correspondences.

**Table 2:** **Meaning of basic notions of frequent item set mining**.

**Mathematics**	**Market basket analysis**	**Spike train analysis**
Item	Product	Neuron
Item base	Set of products	Set of neurons
Transaction *id*	Customer	Time bin
Transaction	Set of products bought by a customer	Set of neurons firing in a time bin
Frequent	Set of products frequently	Set of neurons frequently
item set	bought together	firing together

A transaction 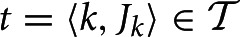 is said to support a subset *J* ⊆ *I* if *J* ⊆ *J*_*k*_. The number of transactions in 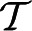 that support *J* is called the *support* of *J* in 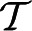 and is denoted by 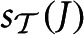 (or simply by *s*(*J*) whenever 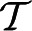 is clear from the context). Frequent item sets are defined based on some user-specified threshold *s*_min_: *J* is called *frequent* in 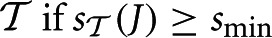.

The FIM problem consists in finding all subsets of *I* that are frequent (in our context: all sets of neurons *A* ⊆ *N* that show frequent synchronous emission of spikes, that is, a number of spike-time coincidences at least *s*_min_). The search for all such item sets exploits that the support operator *s* is *anti-monotone* : for *J*_1_, *J*_2_ ⊆ *I* and *J*_1_ ⊆ *J*_2_ we have *s*(*J*_2_) ≤ *s*(*J*_1_). As a consequence, if *s*(*J*_1_) < *s*_min_ we also know *s*(*J*_2_) < *s*_min_. In words: *no superset of an infrequent item set can be frequent*. This statement is also known as the *Apriori property*.

**Figure 2:**
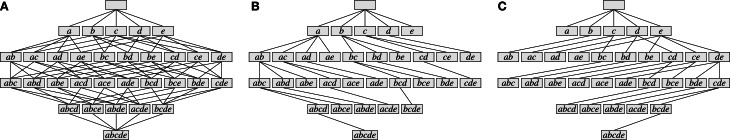
**The search space for five neurons *N* = {*a, b, c, d, e*}. (A**) Hasse diagram: a graph in which each possible set is a node and any two sets *I, J* ⊆ *N* with *I* ⊂ *J* are connected by an edge if and only if ∄*K*: *I* ⊂ *K* ⊂ *J*. **(B)** A selection of edges from the Hasse diagram that reduces it to a search tree. Parents are assigned based on the alphabetical order of the neuron identifiers according to parent(*I*) = *I* \ {max(*I*)}. **(C)** An alternative selection of edges from the Hasse diagram that also reduces it to a search tree. Parents are assigned based on the alphabetical order of the neuron identifiers according to parent(*I*) = *I* \ {min(*I*)}.

The search space is 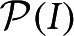, the power set of *I* (that is, the collection of all subsets of *I*). 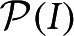 together with the subset relations between its elements is a partially ordered set, which is conveniently represented as a Hasse diagram (see Figure 2A). The search through 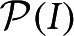 is made irredundant by assigning a unique parent to each item set, which turns the search space into a tree (see Figures 2B,C for two variants). In such a search tree every item set can be reached on exactly one path and therefore it is visited *at most once* in the search. Details about efficient algorithms and data structures to actually carry out the search can be found, for example, in Goethals (2010) and Borgelt (2012).

**Figure 3:**
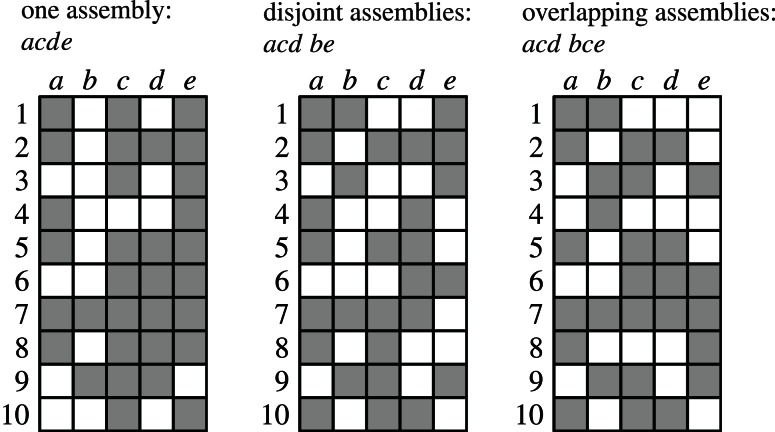
**Parallel spike trains with five neurons *a, b, c, d, e* and 10 time bins containing different neural assemblies**.

**Figure 4:**
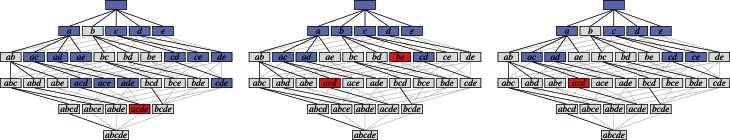
**The search for the data sets shown in Figure 3 illustrated with search trees.** Assemblies are shown in red, all subsets of the assemblies are shown in blue, demonstrating that there always exists a path that only visits frequent subsets.

As an illustration, Figure 3 shows three data sets with distinct neural assemblies. Figure 4 shows, in the search space structured as a tree according to Figure 2B, the frequent item sets that can be found in these data sets for *s*_min_ = 3 (blue and red nodes). If, in analogy to Accretion, only the *maximal frequent item sets* are reported (i.e., no superset is frequent), FIM yields the sets in the nodes marked in red. Note that the search can be pruned with the Apriori property (that is, no supersets of infrequent item sets are explored) without affecting the result: all frequent item sets can still be reached from the root.

## 5. Avoiding redundant search

**Figure 5:**
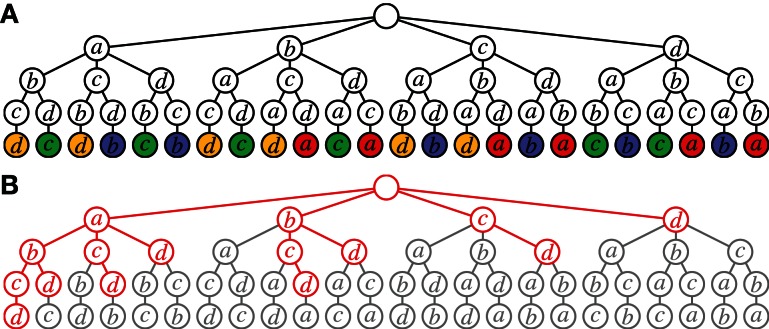
**Redundancy in Accretion. (A)** Unconstrained search for an assembly of four neurons. In nodes with the same color the same statistical test (leaf against path) is carried out. **(B)** Accretion search (whole tree) vs. FIM search (red). The red part corresponds to Figure 2B, the red and gray part together to Figure 2A (all possible paths to the set {*a, b, c, d*} in the Hasse diagram are spelled out in this tree).

As explained above, Accretion derives sequences of neuron ids composing spike patterns rather than sets, which we are actually interested in. As a consequence, it suffers from considerable redundancy. This is demonstrated in Figure 5A, which shows the search carried out for a data set with four neurons if all tests yield significant results. The same set of all four neurons is considered 4! = 24 times (since the search tree has 24 leaves). Even worse, the same statistical test is executed multiple times: leaves having the same color correspond to the same test (the leaf neuron is tested against the *set* of neurons on the path leading to it).

Clearly, this redundancy stems from the fact that the Accretion test ignores the order of the already accreted neurons for the test. Note that it is the main reason why Accretion still needs a branching restriction even with modern computer hardware: without it, an assembly with *n* neurons is considered *n*! times, which becomes infeasible already for moderately large *n*.

However, even if we ensure that each test is executed only once, the same set of *n* neurons is still considered *n* times (four times in Figure 5A, corresponding to the four colors). Although the tests differ, it would suffice to consider each set only once (executing *n* tests on it if desired). In contrast to this, FIM works on sets and with the search space structured as a tree (see Figure 2B or Figure 2C), it guarantees that each set is visited *at most once* (no redundancy). The potential improvements are illustrated in Figure 5B, which show the search space of Accretion for four neurons. Marked in red is the part that is (potentially) explored by FIM (cf. also Figure 2B).

## 6. Criteria for assembly detection

FIM prunes the search and selects results based only on the support of item sets. As a consequence, it may produce results that are not considered by Accretion. For example, a set of neurons may be frequent simply because the member neurons have many spikes and thus are likely to exhibit (non-significant) synchronous spiking. The statistical tests in Accretion eliminate such cases. However, various forms of *statistical testing* can fairly easily be added to FIM as a further evaluation criterion, because the support values of different item sets are all that is needed to compute the test statistics.

In addition, Accretion requires a path of significant test results, which we described above in terms of *subset conditions*. However, such subset conditions may also be added to FIM and we study several variants below.

### 6.1. Statistical testing

Accretion's sequence-based scheme fixes the test to be carried out: the neuron added last is tested against the set of already accreted neurons. In FIM, since each set is considered at most once, we have a choice of *n* tests, where *n* is the number of neurons in the set: each neuron may be tested against the *n* − 1 other neurons. Note that Accretion carries out these tests in different branches of the search tree. Note also that in FIM's search tree (cf. Figure 2B or Figure 2C) there is also a neuron added last, but this neuron depends on an essentially arbitrary global order of the neurons and thus should not determine the test.

Most naturally, we should use an *aggregate* of the results of the *n* tests to evaluate a set of *n* neurons. Following the general principle of statistics to consider the *worst case*, it is most appropriate to use the *maximum* of the *p*-values of the *n* tests. That is, a set of *n* neurons is judged to exhibit significant synchrony if the largest *p*-value of the *n* tests does not exceed the chosen significance level α. In other words, the “most independent” neuron determines the evaluation of the set. Note that Accretion effectively evaluates a set rather by the “least independent” neuron, because a set of neurons is already reported if *one* of the search tree paths leading to it yields a significant test result. That is, Accretion uses the *minimum* of the *p*-values, thus considering, in a certain sense, the *best case*. Note also that Fisher's method of combining *p*-values bearing on the same overall hypothesis (Fisher, 1925) is an alternative to taking the maximum.

Apart from this adapted test procedure, we may ask whether Pearson's χ^2^ independence test is the most appropriate choice. This test assumes that the (discrete) probability of observed frequencies (in the contingency table) can be approximated by the (continuous) χ^2^ distribution. Due to the approximation, some error is introduced, which may lead to incorrect test decisions. The approximation error is generally the larger, the lower the number of degrees of freedom, which is only one for our case of a 2 × 2 contingency table. In addition, for such a table an acceptable approximation requires that all expected counts are at least 5. This renders the test not very well suited for rare events like spike coincidences. As a consequence, it is worthwhile to look for alternatives, which include:

- **Yates's correction for continuity** (or Yates's χ^2^ test).
  Replace the χ^2^ value of Pearson's test with
  $${\text{χ}}_{\text{Yates}}^{2}(A,B)=n_{**}\frac{{\left(|n_{1*}n_{*1}-n_{**}n_{11}|-0.5n_{**}\right)}^{2}}{n_{1*}n_{0*}n_{*1}n_{*0}}.$$
  This correction increases the *p*-value and thus prevents an overestimation of significance, especially if the contingency table has a cell with an expected count less than 5.
- **G-statistic** (or *G*-test).
  A likelihood-ratio based statistical significance test that replaces the χ^2^ value of Pearson's test with
  $$G(A,B)=2n_{**}\sum_{i\;=\;0}^{1}\sum_{j\;=\;0}^{1}\ln\frac{n_{ij}}{n_{i*}n_{*j}}.$$
  The *G*-statistic achieves a better approximation to the theoretical χ^2^ distribution, especially if *n*_11_n_**_ > 2*n*_1*_n_*1_.
- **Fisher's exact test**.
  Compute the *p*-value by summing over the (exact) probabilities of contingency tables with the same marginals that are at least as extreme as the actual table. Although there are alternatives, we rely on the most common choice that “at least as extreme as” means “at most as probable as.” This test avoids approximation, but is costly to compute.

Note that for both Yates's test and the *G*-test the test decision is made (as for Pearson's test) with a χ^2^ distribution with one degree of freedom. That is, the *p*-value is defined by the relations χ^2^_1, 1−*p*_ = χ^2^_Yates_(*A, B*) and χ^2^_1, 1−*p*_ = *G*(*A, B*), respectively.

### 6.2. Subset conditions

Since support is anti-monotone (and thus the Apriori property holds), FIM guarantees that all sets of neurons exhibiting at least *s*_min_ spike coincidences are explored. Accretion's search, however, is guided by statistical test results (or the underlying *p*-values), which do *not* have this property: if a set of neurons does not exhibit significant synchronous spiking, we have no guarantee that there are no supersets that are significant. The *p*-value of an independence test on a superset may be higher or lower than the *p*-value of a test on the set (that is, *p*-values are neither monotone nor anti-monotone). Nevertheless, Accretion does not explore any such supersets, which can reduce the results. Above (in section 3), we formalized this behavior by *subset conditions* that a set of neurons has to satisfy in order to be regarded a *synchronous group*.

Since the FIM search does not impose any such subset conditions, we are free to explore alternatives:

- **No subset conditions.** A set *A* ⊆ *N* of neurons is a synchronous group if all subsets *B* ⊂ *A* with |*B*| = |*A*| − 1 show significant synchrony with the remaining neuron in *A*, that is, with *A* \ *B*. In other words, *A* passes the statistical test described above (and, of course, *s*(*A*) ≥ *s*_min_).
- **Weak subset conditions.** A set *A* ⊆ *N* of neurons is a synchronous group if it satisfies the condition of the first point and, for a user-specified minimum set size *r*, satisfies either |*A*| ≤ *r* or, if |*A*| > *r*, that *at least one* subset *B* ⊂ *A* of cardinality |*B*| = |*A*| − 1 is a synchronous group.
- **Strong subset conditions.** A set *A* ⊆ *N* of neurons is a synchronous group if it satisfies the condition of the first point and, for a user-specified minimum set size *r*, satisfies either |*A*| ≤ *r* or, if |*A*| > *r*, that *all* subsets *B*⊂ *A* of cardinality |*B*| = |*A*| − 1 are synchronous groups.

**Figure 6:**
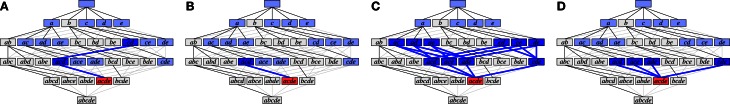
**Illustration of different types of subset conditions (cf. Figure 4A). (A)** Weak subset conditions down to pairs; **(B)** Weak subset conditions down to triplets; **(C)** Strong subset conditions down to pairs; **(D)** Strong subset conditions down to triplets.

Figures 6A,B illustrate weak subset conditions in the FIM search space for the set {*a, c, d, e*} (in red) for *r* = 2 and *r* = 3, respectively. In Figure 6A we have a path from {*a, c, d, e*} down to the pair {*c, d*} (dark blue) and, in Figure 6B, down to the triplet {*a, c, d*} (dark blue). Weak subset conditions mean that there *exist* such paths containing only synchronous groups.

Figures 6C,D illustrate strong subset conditions in the FIM search space for the set {*a, c, d, e*} (in red) for *r* = 2 and *r* = 3, respectively. In Figure 6C we have paths from the set {*a, c, d, e*} down to all pairs contained in it (dark blue) and, in Figure 6D, down to all triplets contained in it (dark blue). Strong subset conditions mean that *all* such paths contain only synchronous groups (whereas weak only asks for at least one path).

Note that weak subset conditions, for *r* = 2, are in fact very similar to Accretion's subset conditions, because Accretion explores a certain sequence only if all of its prefixes produced significant test results. These prefixes form the path required by weak subset conditions. The difference is, of course, that Accretion only requires the one test with the last added neuron to be significant, while we require of a synchronous group that *all* tests of single neurons against all others are significant.

### 6.3. Maximal and closed synchronous groups

Following Accretion, we may report as (candidate) neural assemblies the *maximal synchronous groups*, that is, those synchronous groups for which no superset is a synchronous group. However, such a choice may lose valuable information: if the data contains a significant number of synchronous spiking events of a neural assembly *A*, it is not unlikely that by chance a neuron *a* ∉ *A* produces a spike at a few of these events. This may render the set *A* ∪ {*a*} a synchronous group (if the number of accidentally synchronous spikes is large enough, for which 2 or 3 spikes may already suffice—see experiments below). In such a case, the subset *A* exhibits (many) more synchronous spiking events, which are not considered if only *A* ∪ {*a*} is reported as the maximal synchronous group.

This problem can be addressed by drawing on a notion that is well known in FIM, namely so-called *closed frequent item sets*. A frequent item set is called *closed* if no superset has the same support (while it is *maximal* if no superset is frequent). Since it is unlikely that accidentally synchronous spikes of a neuron *a* ∉ *A* occur together with *all* synchronous spiking events of an assembly *A*, a restriction to closed sets still reports the actual assembly *A*. Therefore, we may report *closed synchronous groups*, which are synchronous groups no superset of which is a synchronous group with the same support. Note that we do not lose anything in this way, because all *maximal synchronous groups* are obviously closed.

Note also that closed synchronous groups avoid certain unintuitive effects of changing the minimum support *s*_min_: while increasing the minimum support may render certain sets maximal which were not maximal before (as supersets may be suppressed), it only eliminates closed sets with a lower support.

## 7. Experimental results

We report results for our assembly detection methods on two types of data: independent parallel spike trains (to check for *false positives*) and parallel spike trains with correlated subsets of neurons (to check for *false negatives*):

- *Independent spike trains*. 1000 trials, each with 100 parallel spike trains (|*N*| = 100), were generated independently as Poisson processes with a constant rate of 20 Hz. The duration of all trials was 3 s and the length of the time bins was 3 ms (1000 time bins).
- *Correlated spike trains*. Trials that contain potential assemblies were generated by injecting synchronous spikes for a subset of the neurons into 100 parallel spike trains, which were independently generated as Poisson processes with a constant rate of 20 Hz for the neurons outside the subset and a constant rate of 20 Hz minus the rate of the synchronous spikes for the neurons in the selected subset. The size *z* of the neuron subset was varied from 2 to 9, the number *c* of injected synchronous spiking events also from 2 to 9, and thus a total of 64 pairs 〈*z, c*〉 were tested. For each pair 1000 trials were generated (3 s duration, 3 ms time bin length, i.e., 1000 time bins), resulting in a total of 64,000 data sets with injected assemblies.

On these data sets we compared Accretion and different FIM-based approaches, namely with no subset conditions as well as weak and strong subset conditions down to pairs (that is, *r* = 2). For each approach we tried all of the statistical tests considered above: Pearson's χ^2^-test, Yates's test, *G*-test and Fisher's exact test. In addition, we executed FIM without any statistical test (and thus without any subset conditions). That is, the minimum support *s*_min_ was the only selection criterion, for which *s*_min_ = 2 was generally chosen (including the methods with subset conditions and statistical tests).

**Figure 7:**
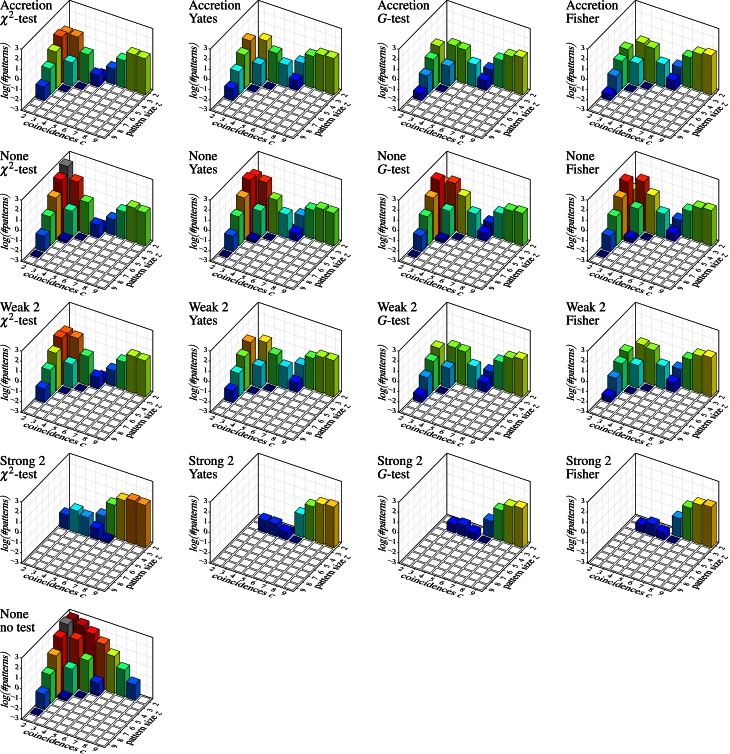
**Independent spike trains:** decimal logarithm of the average number of (significant) patterns found in 1000 trials (white squares: zero patterns, gray bars: higher than chart). Each row of the chart grid corresponds to a detection method (diagram titles, top line; “None”, “Weak 2” and “Strong 2” refer to subset conditions used with FIM, *r* = 2), each column to a statistical test (diagram titles, bottom line). The diagram at the left bottom shows the result of pure FIM (no subset conditions, no statistical test, *s*_min_ = 2).

Figure 7 shows the results on independent spike trains in the form of pattern spectra [like those used for spatio-temporal spike patterns in Gerstein et al. (2012)]. Each bar chart refers to a detection method (Accretion or FIM with different subset conditions; row of the chart grid) and a test statistic (column) and shows the decimal logarithm of the average number of detected patterns (in the sense of *maximal synchronous groups*) subdivided according to the size *z* of the group of neurons underlying the pattern (number of contained neurons) and the number *c* of coincident spiking events (number of time bins with a spike from all neurons in the group). For comparisons, a further bar chart at the bottom shows the result of applying FIM without any subset conditions nor statistical testing (that is, simply an average count of all maximal frequent item sets).

Since the data was generated as independent Poisson processes, all detected patterns are clearly *false discoveries* or *false positives*. The number of such false discoveries is fairly high (note that due to the logarithmic scale, 1, 2, and 3 on the vertical axis stand for 10, 100, and 1000 patterns, respectively, *per trial*). Even fairly pronounced cases like three neurons with five coincident spiking events or five neurons with three coincident spiking events occur in several of the trials. The alternative test statistics (Yates, *G*-test and Fisher instead of Pearson's χ^2^) reduce the number of false discoveries (as expected, because they are less prone to overestimating significance), but are far from solving the problem, especially, since they are effective mainly for small patterns with few coincidences. Subset conditions have a similar effect: weak subset conditions achieve essentially the same result as original Accretion (as expected, see above) and thus suppress some of the false discoveries made without them. However, only strong subset conditions are able to bring the false discoveries down to an acceptable level (assuming that pair patterns are generally ignored).

**Figure 8:**
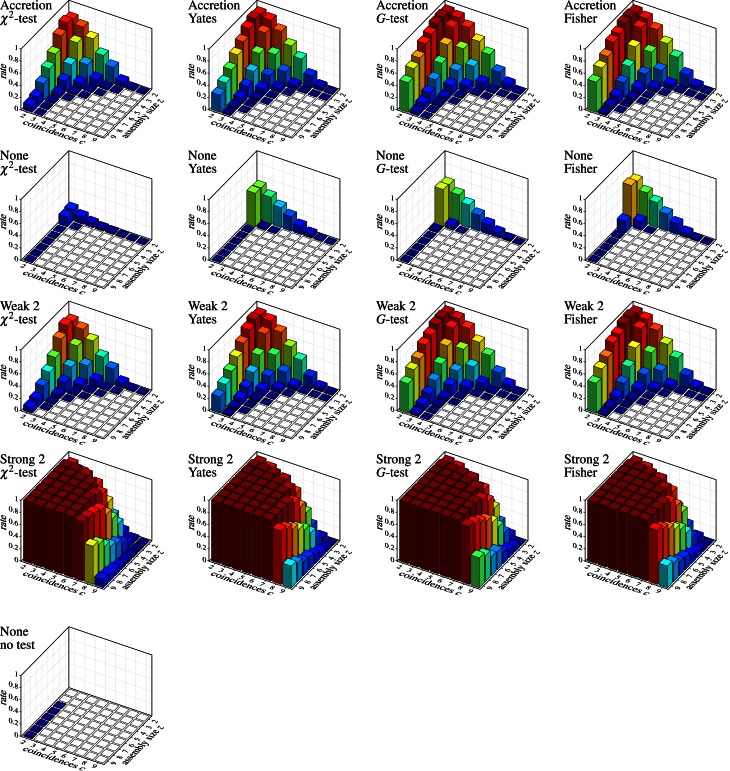
**Correlated spike trains:** false negative rates over 1000 trials, each with a specific injected pattern, characterized by an assembly size *z* and a number *c* of injected coincidences. Rows and columns of the chart grid as in Figure 7. An injected neural pattern is counted as *not* detected (*false negative*) if it is *not* contained in any of the *maximal synchronous groups* produced by the used detection method. Pure FIM (see diagram on the left) necessarily always detects all injected patterns perfectly, unless they are reduced to less than two coincidences by “clipping” caused by the time binning.

Unfortunately, strong subset conditions are no solution either, because they almost prevent discoveries altogether, even correct ones. This can be seen in Figure 8, which shows the results on correlated spike trains as the rate (computed over 1000 trials) of *false negatives* for all detection methods, again subdivided according to the group size *z* and the number *c* of (injected) coincidences. (Note that the actual number of coincidences in the data may be higher, because the injected coincidences may be supplemented by accidental coincidences, but also lower, due to “clipping” caused by the time binning.) An injected pattern is counted as a *false negative* if it is not contained in any of the *maximal synchronous groups* reported by a method. That is, it *is* counted as detected even if it is not exactly among the reported patterns, but only a superset (with additional neurons) has been discovered.

**Figure 9:**
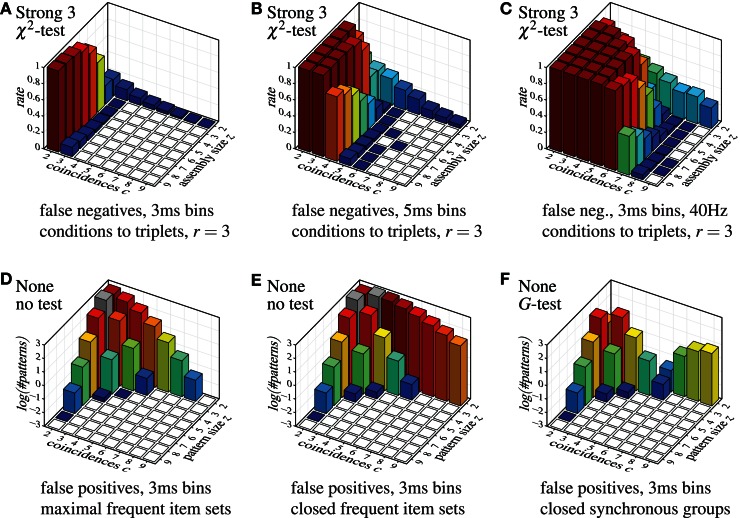
**Selected other false positive and false negative results,** demonstrating the effect of the parameter *r* (that is, the lower size limit for the subset conditions), the effect of the time bin width and the firing rate in combination with strong subset conditions down to triplets, and the effect of using closed instead of maximal sets or synchronous groups. Note the logarithmic scale (decimal logarithm) of the vertical axis of the false positive bar charts.

Despite this somewhat lax criterion, FIM with strong subset conditions is hardly able to detect the injected patterns, unless a not too large assembly (fairly few neurons) exhibits a fairly large number of coincidences. Even clear cases like eight neurons firing together seven times are not detected. The reason, however, is obvious: with strong subset conditions and *r* = 2, all pairs contained in a group of neurons have to test positive in order for the group to qualify as a pattern. The more neurons there are in a group, the more pairs there are and thus the more likely it is that one of them is, by accident, not significant, unless the number of (injected) coincidences is fairly large. The situation is improved with *r* = 3, but the detection rate still suffers for larger assemblies (see Figure 9A), and the more so if we consider larger time bins (Figure 9B). Furthermore, higher firing rates worsen the situation as well (Figure 9C).

Accretion and FIM with weak subset conditions exhibit better detection capabilities, but are far from impressive either. Only if we abandon subset conditions, the detection rate is significantly improved (few false negatives). Note that FIM without subset conditions and relying only on minimum support (no statistical tests) necessarily detects all injected patterns, unless the coincident spiking events are reduced to less than two by “clipping” due to the time binning.

In general, the tougher the subset conditions of the assembly detection method (including a lower value of *r*) the lower the number of false positives, but the higher the rates of false negatives. Alternative statistical tests have a similar effect: Yates's test, *G*-test and Fisher's exact test reduce the number of false positives, but at the price of higher rates of false negatives. The same holds for the significance level (not shown, but obvious): the lower α, the fewer false discoveries are made, but at the price of fewer correct discoveries (more false negatives). If we take into account what false positives are suppressed (by whatever approach), namely mainly small patterns with few coincidences (back corners of the bar charts), while larger patterns with more coincidences are essentially unaffected, one may wonder whether statistical tests and subset conditions are actually worth the effort (because both increase the computation time considerably compared to a pure FIM approach).

Generally we can say that the core problem of a reliable detection of neural assemblies is the large number of false positives, which the discussed methods cannot reduce without severely harming the detection sensitivity of the method. This number of false discoveries may appear to be surprising at first sight, because we used a significance level of α = 1%. However, this applies only to an *individual test*, whereas we are executing a huge number of tests in the search. That is, we face the problem of *multiple testing*, due to which we lose an effective control over the significance level. In simple terms: if we execute 1000 (independent) tests with α = 1%, we should expect about 1000 · 1% = 10 positive test results, simply as chance events, signifying nothing. In our search, however, we even execute millions of tests. For example, 100 recorded neurons allow for $\left(\begin{array}{c} 100\\ 3 \end{array}\right)=161,700$ triplets and $\left(\begin{array}{c} 100\\ 4 \end{array}\right)=3,921,225$ quadruplets. As a consequence, even though it is very unlikely that, say, four *specific* neurons fire together three times if they are independent (such an event has a *p*-value of less than 10^−6^ in our experimental setup with the testing methodology described above), it is fairly likely that we observe *some set* of four neurons firing together three times. Indeed, in our experiments we see, on average, more than one such pattern per trial.

Unfortunately, because of the excessive number of tests executed in the search, standard methods to handle the multiple testing problem (the like *Bonferroni correction* (Bonferroni, 1935; Abdi, 2007), the *Holm-Bonferroni method* (Holm, 1979), or the *Benjamini-Hochberg procedure* Benjamini and Hochberg (1995); see Dudoit and van der Laan (2008) for an overview) require *p*-values so low that they are extremely unlikely to be obtained from actual data, and also lead to a very high rate of false negatives: effectively we cannot expect to make *any* discoveries anymore. As a consequence, we have to change the testing methodology in order to obtain a suitable method (see the next section).

Finally, the diagrams in Figure 7 show that a detection based on *maximal synchronous groups* causes strange effects: while pure FIM reports no pairs with 9 coincidences and only few with 8, all other methods detect several such pairs (or at least more than pure FIM). The reason is that these patterns are, of course, present in the data, but they are not *maximal frequent item sets*. These patterns rather have supersets with three or four neurons (exhibiting fewer coincidences), which are reported by pure FIM and thus eliminate the pairs. These supersets are suppressed, though, by the statistical tests or the subset conditions of the other detection methods, rendering the pairs maximal. Note that these patterns are found with pure FIM if we report the *closed synchronous groups* (closed frequent item sets) as demonstrated in Figure 9E as compared to Figure 9D (which repeats the bottom left bar chart of Figure 7). Note that the pattern counts are affected the less (by reporting closed patterns instead of maximal) the larger the patterns, even if a statistical test is used (see Figure 9F for an example).

## 8. Assembly detection with FIM

Based on the insights gained in the previous section, we propose now an assembly detection method based on (pure) FIM that reduces the problem of multiple testing considerably. In this method we no longer look at specific neuron groups, which is the main reason for the huge number of tests. Rather we rely on the rationale that a pattern of a certain size *z* and exhibiting a certain number *c* of coincident spiking events cannot reliably identify an assembly if a counterpart—that is, a neuron group with the same size *z* (but possibly different composition) and the same number *c* of coincidences—occurs in properly generated surrogate data (rendering the neurons independent). The reason is that the occurrence of a counterpart in surrogate data demonstrates that the pattern could be a chance event and thus that it is not significant. To be more specific, our assembly detection method works as follows:

- *FIM on Original Data*. We apply FIM to our original (binned) data and report all *closed frequent sets* of neurons together with their support, where a neuron set is called *closed* if all of its supersets have a lower support. We prefer closed over maximal sets due to the reasons pointed out above, especially the loss of support information incurred by reporting only maximal sets. We recommend *s*_min_ = 2, but higher values may also be used.
- *FIM on Surrogate Data*. In order to determine which closed frequent neuron sets found in the original data may be due to chance events, we generate surrogate data sets. That is, we create modifications of the original data that are intended to keep all of their essential features except spike synchrony—the feature we are testing for. [For a survey and analysis of the methodology to generate surrogate data from parallel spike trains, see, for example, (Grün, 2009; Louis et al., 2010b,c)]. To each surrogate data set we apply FIM and collect the *closed frequent sets* together with their support. More specifically, we collect the signatures 〈*z, c*〉 (size *z* and coincidences *c*) of found patterns. Afterward we eliminate from the closed frequent neuron sets found in the original data all sets for which a counterpart (that is, same size *z* and same number *c* of coincidences, but possibly different composition, that is, different underlying set of neurons) was found in a surrogate data set, since such sets could be chance events (see below for a more detailed justification and discussion of this procedure).

Note that this procedure still suffers from a certain amount of *multiple testing*: every pair 〈*z, c*〉 that is found by FIM in the original data gives rise to one test. However, the pairs 〈*z, c*〉 are *much fewer* than the specific neuron sets that are considered in all methods discussed above. As a consequence, simple approaches like *Bonferroni correction* (Bonferroni, 1935; Abdi, 2007) become feasible. That is, we divide the desired overall significance level α by the number *n* of tests to obtain the significance level α′ = α/*n* for each individual test. Since in practice we can expect to find only a few dozen pairs 〈*z, c*〉 in the data to analyze, we obtain significance levels α′ that leave us good chances of making detections. The number *n* of tests (that is, 〈*z, c*〉 pairs) may even be reduced further by the insight that patterns with signatures like 〈2, 2〉, 〈3, 2〉, 〈2, 3〉 etc. are certainly discovered in the data, but we do not consider these patterns as candidates for assemblies right from the start. Only pairs 〈*z, c*〉 with sufficiently large *z* and/or *c* need to be counted, for which we are actually willing to accept the underlying neuron sets as assemblies.

Technically, the significance level α enters the testing procedure as the number of surrogate data sets to be generated. For example, if we choose α = 1% and there are *n* = 30 pairs 〈*z, c*〉 in the original data which we actually want to submit to a test, we have to generate *k* = *n*/α = 3000 surrogate data sets. The reason is that of the results on the original data we keep only such neuron sets for which we do *not* see a counterpart in any of the surrogate data sets. That is, if we do not observe any occurrence of a specific pair 〈*z, c*〉 in *k* = 3000 surrogate data sets, then we can estimate the probability of this pair 〈*z, c*〉 occurring by accident as less than *p* = 1/*k* = 1/3000. This is the *p*-value of the test and since it is *p* ≤ α′ = α/*n* = 0.01/30 = 1/3000, any pattern with the signature 〈*z, c*〉 is significant.

Note that the resulting procedure is computationally feasible due to the sophisticated high-speed implementations that are available for closed FIM^2^. Even running FIM on thousands of surrogate data sets takes only a few minutes on modern computer hardware. In addition, this process can easily be parallelized, since the surrogate data sets can be processed independently, while collecting the 〈*z, c*〉 pairs from all surrogate data sets takes negligible time.

**Figure 10:**
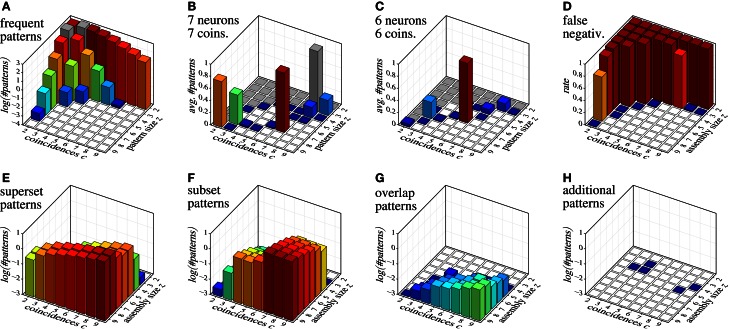
**Assembly detection with surrogate data filtering based on size/coincidence signatures 〈*z, c*〉. (A)** decimal logarithm of the average number of patterns found in 10,000 surrogate data sets; **(B and C)** average numbers of patterns detected in 1000 data sets with injected assemblies with 〈*z, c*〉 = 〈7, 7〉 and 〈*z, c*〉 = 〈6, 6〉, respectively (gray squares indicate signatures that occur in the surrogates and are therefore eliminated); **(D)** false negatives for injected assemblies with different 〈*z, c*〉 combinations; **(E–H)** decimal logarithm of the average number of patterns with different relations to the injected assembly: **(E)** proper supersets of the injected assembly, **(F)** proper subsets of the injected assembly, **(G)** patterns with at least two neurons from the injected assembly and at least one other neuron, **(H)** patterns having none or at most one neuron in common with the injected assembly.

As an example of detecting assemblies with this procedure, we consider a trial with 100 parallel spike trains with an injected assembly of seven neurons (labeled {1, 2, 3, 4, 5, 6, 7}) and 7 coincidences, generated as described in section 7 for correlated data (20 Hz firing rate, duration 3 s, time bin length 3 ms, that is, 1000 time bins). We generate 10,000 surrogate data sets by means of spike-time randomization (see, e.g., Louis et al., 2010c; this simple approach is acceptable here due to the stationary process). The average number of closed patterns found with FIM in these surrogate data sets, subdivided by their size *z* and number *c* of coincidences (support) is shown in Figure 10A. After removing from the result on the original data all closed sets with signatures 〈*z, c*〉 that occur in surrogate data, we obtain the sets shown in Table 3.

**Table 3:** **Closed frequent neuron sets found in data with an injected assembly with seven neurons and seven coincidences, after filtering with surrogate data; support/coincidences after the colon**.

{1, 2, 3, 4, 5, 6, 7, 81, 85} : 2	{2, 3, 5, 6} : 8	{2, 3, 7} : 8
{1, 2, 3, 4, 5, 6, 7, 35} : 4	{2, 3, 4, 35} : 5	{2, 5, 7} : 8
{1, 2, 3, 4, 5, 6, 7, 93} : 3	{2, 3, 6} : 9	{3, 4, 7} : 8
{1, 2, 3, 4, 5, 6, 7} : 7	{2, 3, 4} : 8

This is a fairly typical result (although it was specifically selected to cover all relevant effects): the injected assembly is detected, but also some other sets, which, however, are all related to the injected assembly. Due to chance coincidences of neurons outside of the assembly (here: neurons 35, 81, 85, 93) with some of the coincident spiking events of the assembly, we see a few supersets of the assembly, with lower support. Due to chance coincidences (resulting from background spikes) of some of the assembly neurons, we also see subsets of size 3 and 4, with a support exceeding the injected coincidences by 1 or 2. Finally, we see a set overlapping the assembly ({2, 3, 4, 35}), which results from neuron 35 firing together with four coincident spiking events of the assembly and the one additional coincident spiking event of the neurons {2, 3, 4} (which is caused by background spikes). Note that there are no sets that are unrelated to the injected assembly. How this result set can be reduced to the (most likely) assembly will be the topic of a subsequent paper (see Torre et al., in preparation).

However, compared to, say, Accretion, this result is already a huge improvement: running Accretion with a branching restriction of 2 on the same data yields (after removing duplicates—recall the redundancy of Accretion's search scheme) 66 sets, only 7 of which are related to the injected assembly (3 are supersets, 4 are overlapping patterns), while the assembly itself is not in the output. The unrelated patterns have sizes ranging from 2 to 5 neurons and exhibit between 2 and 9 coincidences. Without a branching restriction, Accretion yields even 105 sets, 30 of which are related to the injected assembly (5 supersets, 25 overlapping patterns).

In order to illustrate the detection capabilities of our method, Figure 10 collects various results for data sets with injected assemblies with different sizes *z* and coincidences *c*. Figure 10B shows the average number of patterns that are detected in data sets with an injected assembly with seven neurons and seven coincidences (averages over 1000 runs). Figure 10C shows analogous results for six neurons and six coincidences. Figure 10D shows the false negative rate (fraction of runs in which neither the assembly itself nor a superset is in the result set) for individual injected assemblies, covering all possible 〈*z, c*〉 combinations in {2,…,9}^2^ (averages over 1000 runs). Note that false negatives occur basically only for 〈*z, c*〉 combinations that occur in surrogate data (see Figure 10A). Clearly, this is unavoidable.

The few false negatives represented by the blue squares are due to “clipping” caused by the time binning, which reduces the injected number of coincidences and thus creates a 〈*z, c*〉 pair that occurs in surrogate data. Note that for some signatures that occur in the surrogate data (e.g., 〈*z, c*〉 = 〈8, 2〉 and 〈*z, c*〉 = 〈3, 7〉) the injected assembly *is* sometimes detected, even though these signatures occur in surrogate data. The reason are chance coincidences, either of the whole set (for small *z*) or of an additional neuron with all coincident spiking events (for small *c*), which creates a pattern with a signature that is *not* eliminated by surrogate filtering.

Figures 10E–H give an idea of what and how many patterns are to be expected in the result for data with a single injected assembly, subdivided according to the size *z* and the coincidences *c* of the assembly. Not surprisingly, supersets become more frequent with an increasing number *c* of coincident spiking events, subsets more frequent with an increasing size *z* of the injected assembly. Overlapping patterns are much fewer (note the logarithmic scale!) and their number grows with both the size *z* and the coincidences *c*. A clear benefit of our method is that it almost never produces patterns unrelated to the injected assembly (see Figure 10H): in the total of 64,000 data sets that the bar charts represent (1000 data sets per bar), only five patterns were detected that were *not* related to the assembly.

## 9. Discussion

We started this paper by reviewing and analyzing the so-called Accretion method (Gerstein et al., 1978) for identifying neural assemblies from (discretized) parallel spike trains. Inspired by Accretion, we presented alternative assembly detection methods built on modifications and/or refinements of the two main constituents of Accretion: the statistical test to determine significance of the neuronal patterns built in the process and the subset conditions that a set of neurons needs to satisfy in order to be considered a neural assembly. Subset conditions alternative to those of Accretion were implemented with the help of FIM. By working on sets instead of sequences in a tree-like search space, FIM overcomes Accretion's redundancies and proves to be more efficient: it yields shorter execution times even though the search is actually exhaustive, because no branching restriction is employed.

The results of our tests on trials with both independent spike trains (generated as independent Poisson processes) and correlated spike trains (generated as independent Poisson process with injected spike coincidences) showed high rates of false positives and false negatives for Accretion. Some of the FIM-based models that were built on alternative subset conditions and/or statistical tests performed better in terms of false positives but, generally, any such improvement was paid for by an increase in the number of false negatives. FIM alone, with no subset conditions and no statistical test, is not significantly worse than the other models since, although it yields the largest amount of false positives, it produces essentially no false negatives. Overall, the significance criteria employed by all of these models do not properly take into account the multiple testing problem, since they focus on tests of individual patterns. As a consequence, a high rate of false discoveries (false positives) and/or false negatives is always to be expected.

In the view of such results we proposed an alternative assembly detection method, based solely on FIM, which addresses the multiple testing problem properly. It is based on analyzing (with FIM) surrogate data that is generated from the original spike trains. The (closed) neurons sets that are found (with FIM) in the original data are then filtered by removing all patterns for which a counterpart occurs in surrogate data, that is, for which a pattern with the same size *z* and the same number *c* of coincidences (support) was found in some surrogate data set. The rationale underlying this approach is that a pattern with a counterpart in surrogate data could be a chance event and thus should not be considered significant.

Since we employ sophisticated and high-speed implementations of FIM algorithms to find the closed neuron sets, our method is efficient even though it requires to generate and analyze a substantial number of surrogate data sets. For example, generating and analyzing the 10,000 surrogate data sets underlying Figure 10A takes about 2 minutes on standard modern computer hardware, even without parallelization. This enables us to apply the method in a sliding window fashion in order to follow the dynamics of assembly activity.

This paper presented the basic approach of FIM and relevant statistics to detect and identify spike synchrony patterns in massively parallel spike data. In a subsequent paper (in preparation) we will report about further studies of dependencies on various analysis parameters (e.g., time bin size) and on features of the data [e.g., level of firing rates, various non-stationarities, deviations from Poisson processes etc. (Grün, 2009)]. However, our previous studies in other contexts (e.g., Louis et al., 2010b,c) and preliminary studies of our FIM-based method make us confident that we can account for such aspects by using surrogates that incorporate such features, e.g., local spike dithering or shift-surrogates (Gerstein, 2004; Pipa et al., 2008; Louis et al., 2010b).

Maximum-entropy models recently found application in the context of identification of the correlation structure in parallel spike trains. A general difference of methods based on maximum-entropy models in comparison to our approach is, that they evaluate if correlation structures exist at all by comparing to non-existent correlations, whereas our approach aims to detect specific correlation structures. Although (Schneidman et al., 2006) claims that spike correlations found in experimental data are fully explained by pairwise correlations only Shlens et al. (2006), discusses in its study that existent higher-order correlations may be missed since they contribute only to a small percentage in the explanatory power. Roudi et al. (2009) showed in an extensive study that the estimates of the correlation structure estimated by maximum entropy models strongly depends on the existent parameters, in particular the size of the data set, and thus may lead to wrong conclusions on the correlation structure with a bias toward pairwise correlations. Thus we do not expect that maximum-entropy models would be able to extract assemblies of such small size of neurons and small number of occurrences of correlated spiking within a large number of neurons as it is possible with our approach.

In Feldt et al. (2009) a modification of Accretion was suggested as a way to improve its performance. The idea is to use the *p*-value of an independence test (e.g., Pearson's χ^2^) as the distance between two neurons (or sets of neurons) in a hierarchical agglomerative clustering algorithm. In general, such clustering starts with each of the given objects (here: neurons) in its own cluster. In each subsequent step it greedily merges the two clusters with the smallest distance, provided this distance is no greater than a user-specified maximum distance. For the Accretion variant, distances are measured by the *p*-value of an independence test and thus this maximum distance is simply the chosen significance level α. Once no neuron groups can be merged any more, the method returns all clusters with more than one neuron. This method could be referred to as a “matrix version” of Accretion, because hierarchical agglomerative clustering is usually implemented as operating on a distance matrix, from which the rows (and corresponding columns) of the two clusters (here: sets of neurons) to be merged are removed and replaced by a row (and a column) representing the new cluster that resulted from the merger.

The matrix version of Accretion has the clear advantage that there is no branching. It executes at most *n* − 1 steps for *n* neurons (after which all neurons would be merged into a single cluster) and cannot produce more than ⌊*n*/2⌋ clusters (unless singletons are counted as well). It may also be argued that it sometimes carries out more meaningful statistical tests, because it may merge two clusters both of which already contain two or more neurons, while Accretion always tests an already accreted group of neurons against a *single* new neuron.

However, these advantages are more than equalized by several disadvantages. Although the matrix version generally yields fewer results due to its greedy search scheme, it may still produce many false positives, because the problem of false positives lies mainly in the *potential* number of tests (i.e., the size of the search space) and the nature of the statistical tests and not in the *actual* number of tests that are executed. In addition, since the matrix variant also reports maximal sets, one almost never obtains an injected assembly exactly, but rather a superset (like in standard Accretion). Finally, as a hierarchical agglomerative clustering approach, it necessarily yields disjoint sets of neurons as (candidates of) assemblies. This leads to an unavoidable loss of results in case of overlapping assemblies, but may also rip an assembly apart if a bad merger is chosen due to the data characteristics, which is particularly likely when merging neurons into pairs.

The faster execution of the matrix version of Accretion is certainly attractive, but with the sophisticated, high-speed FIM implementations that are available, which make our method fairly efficient, there is no need to accept any of its drawbacks.

The only feature currently missing from our FIM-based approach is a way to reduce the found pattern set to the (most likely) assembly or assemblies. While a human can easily spot the actual assembly (or assemblies) by looking at the (usually reasonably small) output (see, for example, Table 3), an automatic method is desirable. We are currently working on a paper (Torre et al., in preparation) that presents and compares several suggestions of such pattern set reduction methods, which proved to be highly promising in preliminary experiments.

### Conflict of interest statement

The authors declare that the research was conducted in the absence of any commercial or financial relationships that could be construed as a potential conflict of interest.

## Footnotes

^1^Recall that an *A*-event occurs only if *every* neuron in *A* has a spike in the time bin. That is, “not *A*” means that *not all* neurons in *A* have a spike in the time bin and *not* that *no* neuron in *A* has a spike in the time bin.

^2^See, for example, fimi.ua.ac.be/src or www.borgelt.net/fpm.

## Software and additional material

The FIM library underlying the Python scripts with which we carried out our experiments is available at http://www.borgelt.net/pyfim.html

The actual Python and shell scripts as well as more extensive result diagrams are available at http://www.borgelt.net/accfim.html
